# Support for hospital doctors’ workplace well-being in England: the Care Under Pressure 3 realist evaluation

**DOI:** 10.1136/bmjqs-2024-017698

**Published:** 2025-04-11

**Authors:** Anna Melvin, Alison Pearson, Daniele Carrieri, Charlotte Bramwell, Jason Hancock, Jessica Scott, Helen Foster Collins, Stuart McPhail, Mark Pearson, Chrysanthi Papoutsi, Geoff Wong, Karen Mattick

**Affiliations:** 1 Department of Health & Community Sciences, University of Exeter, Exeter, UK; 2 Department of Public Health and Sport Sciences, University of Exeter, Exeter, UK; 3 Devon Partnership NHS Trust, Exeter, UK; 4 Institute of Clinical & Applied Health Research, Hull York Medical School, University of Hull, Hull, UK; 5 Nuffield Department of Primary Care Health Sciences, University of Oxford, Oxford, UK

**Keywords:** health services research, humaneness, leadership, patient safety, teams

## Abstract

**Introduction:**

The vital role of medical workforce well-being for improving patient experience and population health while assuring safety and reducing costs is recognised internationally. Yet the persistence of poor well-being outcomes suggests that current support initiatives are suboptimal. The aim of this research study was to work with, and learn from, diverse hospital settings to understand how to optimise strategies to improve doctors’ well-being and reduce negative impacts on the workforce and patient care.

**Methods:**

Realist evaluation consistent with the Realist And Meta-narrative Evidence Synthesis: Evolving Standards (RAMESES) II quality standards. Realist interviews (n=124) with doctors, well-being intervention implementers/practitioners and leaders in eight hospital settings (England) were analysed using realist logic.

**Results:**

There were four key findings, underpinned by 21 context-mechanism-outcome configurations: (1) solutions needed to align with problems, to support doctor well-being and avoid harm to doctors; (2) doctors needed to be involved in creating solutions to their well-being problems; (3) doctors often did not know what support was available to help them with well-being problems and (4) there were physical and psychological barriers to accessing well-being support.

**Discussion and conclusion:**

Doctors are mandated to ‘first, do no harm’ to their patients, and the same consideration should be extended to doctors themselves. Since doctors can be harmed by poorly designed or implemented well-being interventions, new approaches need careful planning and evaluation. Our research identified many ineffective or harmful interventions that could be stopped. The findings are likely transferable to other settings and countries, given the realist approach leading to principles and causal explanations.

**WHAT IS ALREADY KNOWN ON THIS TOPIC**

- Despite a growing focus on healthcare workforce well-being, the problem continues.
- Understanding how to support doctors’ well-being at work would benefit doctors, patients and the wider healthcare system.

**WHAT THIS STUDY ADDS**

- There is often misalignment between what doctors perceive to be the problems for well-being and the solutions offered.
- This misalignment is not just neutral but can cause harm to well-being, leading to feelings of frustration, cynicism and being undervalued.

**HOW THIS STUDY MIGHT AFFECT RESEARCH, PRACTICE OR POLICY**

- For interventions to be meaningful and effective, doctors need to be involved in identifying problems and creating solutions relevant to their well-being.

## Introduction

 Poor well-being in doctors has been recognised across healthcare settings globally.^1^ Even prior to the COVID-19 pandemic, doctors’ well-being was in crisis with high levels of burnout, stress, mental ill-health, suicide and substance abuse.^1^,^6^ Despite growing attention, the problem continues, suggesting the current approach is not working well enough to resolve it.^7^ Since 2020, the situation has only worsened, as healthcare systems face increasing pressures and doctors are placed under strain with increasing workloads and feeling unable to deliver high-quality patient care.^8 9^ Doctors face a vicious cycle in which high workload and pressures reduce job satisfaction and increase burnout, leading to more doctors leaving or working fewer hours, which in turn increases workload and pressures.^9^ Stress and well-being are cited as key reasons for doctors delaying taking up specialty training and needing to take a break.^10 11^

The Quadruple Aim of healthcare recognises workforce well-being as integral to achieving the other three aims of healthcare: improving patient experience and population health and reducing costs.^2^ Doctors’ well-being is linked to organisational performance and healthcare service delivery as well as patient care and safety.^12^,^14^ The financial implications are significant. Sickness absence in the National Health Service (NHS) costs £1.1 billion a year,^15^ and additional staffing (agency and bank) costs totalled £9.2 billion in 2021–22.^16^ Training a UK doctor to Foundation Year 2 represents an investment of £327 074 and to consultant level, it is £584 102.^17^ Therefore, alongside moral and ethical imperatives to improve doctors’ well-being, there is also a strong business case.^18^

Previous research explored and explained the complex factors leading to poor well-being, using theories and frameworks from psychology, sociology, education and business among others, but much less is known about how to better implement support for doctors at work. Despite multiple approaches, there is no conclusive evidence about which interventions are effective.^19 20^ This is unsurprising, since with complex problems, different things work in different ways in different settings. We need to understand this complexity and gain nuanced insights to underpin the design of evidence-based solutions that are tailored to settings. There remains a pervasive narrative within medicine emphasising individual resilience and doctors’ responsibility for their own well-being.^5^ However, this ignores system influences in causing well-being problems.^5 21 22^ More research is needed about which interventions work for different doctors and how individual and system interventions can be combined.^23^

Realist research is well suited to the study of complex interventions, since it provides deeper insights into the nature of programmes and implementation contexts. Our previous work, using realist approaches, explored how to improve existing interventions rather than developing new ones.^24^,^26^ Carrieri *et al* concluded that doctors were more likely to experience mental ill-health when they felt isolated, unable to do their job or feared the repercussions of help-seeking.^24 25^ Taylor *et al* observed that it is difficult to promote staff psychological wellness within blame cultures; that system needs often override staff psychological well-being and that interventions tend to be fragmented, individual-focused and insufficiently recognise cumulative chronic stressors.^26^

While there have been growing calls for system-based change, it remains unclear how to enact these due to a paucity of research and implementation guidance.^21 24 25 27^ Therefore, this research used a realist approach to develop context-sensitive causal understandings that can guide implementation in practice.

## Methods

### Aim

To work with, and learn from, eight diverse hospital settings to understand how to optimise strategies to improve doctors’ well-being and reduce their negative impacts on the workforce and patient care. The research question was: What works, for whom, in what circumstances, how and why (not) in supporting hospital doctors’ well-being?

### Study design and terminology

This was a realist evaluation, a form of theory-driven research well suited to complex interventions, since it recognises that what works in one situation may not work in another, and provides deeper insights into the nature of programmes and implementation contexts.^28^ The protocol was published previously.^29^

The term ‘well-being’ referred to broad workplace mental health and well-being experiences, from promoting psychological well-being to supporting those with specific mental health problems.^30^ The term ‘intervention’ referred to a wide range of support offerings, including those that improve workplaces (eg, shared social spaces), those that improve staff resources (eg, stress management training) and those that resolve problems (eg, counselling).^31^

### Study context

The eight hospital settings were called ‘trusts’, which are organisational units within the NHS in England, generally serving either a geographical area or a specialised function. We recruited Acute NHS trusts, which provide secondary care services. The data were collected in 2023–2024, 3 years after the COVID-19 pandemic onset and during a period of doctors’ industrial action regarding pay and conditions.

### Initial programme theory

The initial programme theory (IPT) (table 1) was developed based on previous published research^24^,^2631^ and iterative discussions within the research team.

**Table 1:** IPT, which provides an overarching understanding of and approach to the issue, and informs the development of the final programme theory

IPT area	Examples of content
Wider context	Professional culture affecting doctors’ expectations of their role based on professional norms, and NHS context affecting the conditions within which doctors work.
Workplace context	System pressures placing demands on doctors to deliver care and put patient needs before their own, and culture and leadership affecting the perception of well-being in the trust and interventions offered.
Workplace well-being strategy	The types of interventions offered to support well-being determined by timing, funding structures, responsible persons’ expertise and power to influence senior leadership.
Work environment	The extent to which doctors’ basic needs are met at work (eg, hydration, parking, breaks), psychological safety of work environments and doctors’ relationships with colleagues.
The doctor’s situation and relationship to work	Extent to which doctors feel able to carry out their role, type of role (eg, in a training programme, locally employed, specialty and specialist doctors, consultant) and international medical graduates.

Please note that, according to the RAMESES II standards, initial programme theories may take different formats and may or may not be realist in nature at the outset, since they will be ‘re-cast’ in realist terms through the evaluation process.

IPT, initial programme theory; NHS, National Health Service.

### Data collection

Eight Acute NHS trusts in England were purposively sampled using several data sources (online supplemental appendix 1), as per Emmel’s guidance.^34^ Stakeholders in each trust were engaged throughout the project, to champion the study, facilitate recruitment and provide feedback on analysis. Site visits facilitated recruitment and contextualised the findings.

Based on the IPT, three groups of participants were purposively sampled within each trust (some fitted more than one category).

1. Doctors, as intervention recipients, covering all career stages and employment types (eg, consultants, doctors in training, trust grade/locally employed doctors, specialty and specialist doctors, UK-trained and international medical graduates).
2. Staff with roles supporting doctors, who put well-being strategies into action through making decisions about interventions or delivering interventions (eg, medical education managers, human resources and occupational health staff, counsellors, psychologists, chaplains).
3. Leaders with strategic roles in relation to well-being, who determine trust policies and influence the trust’s culture and well-being strategy (eg, Head of Organisational Development, Directors of Medical Education, Chief Executive Officer, Freedom to Speak Up Guardians).

Participants were recruited through posters, emails, key contacts and snowballing. A Participant Information Sheet was emailed in advance of the interview and informed consent was given at the start.

### Realist interviews

Realist interviews^35^ were undertaken to develop an explanatory account of how different interventions within and across trusts are working, for whom, under what circumstances, how and why (not) and to understand participants’ insights about the different contexts, mechanisms and outcomes that may be important. The topic guide (see online supplemental appendix 2) explored perceptions of well-being, organisational culture and well-being interventions. Development of the topic guide was informed by the aims and objectives of our study (see published protocol),^29^ the IPT (see table 1) and feedback provided by our stakeholder group (see later). We conducted 124 online interviews between 9 May 2023 and 4 January 2024 via Microsoft Teams (12–23 interviews per trust). Participants received a £20 Amazon voucher. Interviews were recorded and transcribed verbatim by professional transcribers. Interviews lasted 47 min on average. Reflective notes written immediately afterwards captured interviewers’ initial thoughts and impressions.

### Data analysis

Interview transcripts were uploaded to NVivo. Data analysis was concurrent with data collection. Each new element of relevant data was used to refine aspects of the programme theory, and as it was refined, data sources were re-scrutinised to search for data relevant to the revised programme theory that may have been missed initially. Guided by the RAMESES II quality standards,^28^ we used a realist logic to make sense of the IPT, building causal explanations in the form of context-mechanism-outcome configurations (CMOcs) for the programme theory. This coding was inductive, deductive (informed by the IPT and substantive theory including self-determination theory, Maslow’s hierarchy of needs and the job demands-resources theory)^36^,^38^ and abductive. Data were first interpreted to ascertain if they pertained to context (C), mechanism (M), outcome (O), the relationships between C, M and O and/or the relationships between CMOcs. Interview notes were considered alongside coding, and repeated rounds of coding and team discussion (often using mind maps) facilitated further insights. We used interpretive cross-case comparison to understand and explain how and why observed outcomes had occurred, for example, by comparing interventions where improving well-being was deemed ‘successful’ in some trusts and not others, to understand how context was influential. We moved iteratively between analysis of particular examples from the data, refinement of programme theory and further testing of subsections of programme theory. We used multiple analytic reasoning processes to make sense of the data: juxtaposing evidence sources; reconciling evidence sources; adjudicating evidence sources and consolidating evidence sources.^39^ To preserve anonymity, we changed some intervention names and removed parts of quotes, indicated by square brackets […].

### Team reflexivity

The multidisciplinary team of experienced researchers and clinicians, with topic area and methodological expertise, brought diverse perspectives (eg, psychological, sociological, educational, clinical, implementation science) to the research. The medically qualified team members came from diverse medical specialties including general practice, psychiatry, anaesthetics and emergency medicine. These aspects of diversity were important given the complexity of the problem of well-being in doctors.

### Stakeholder engagement

Since complex interventions are more likely to be successful when researchers co-create them with practitioners,^40 41^ we were supported by an online stakeholder group, who met regularly to discuss and provide feedback on research processes, findings, outputs and dissemination. Stakeholders included doctors from shortage specialties, doctors who have experienced mental ill-health, other healthcare professionals and managers, patient representatives, doctor support organisations, relevant academic experts and trust representatives.

## Findings

### Characteristics of settings and participants

Key characteristics of the eight trusts are outlined in online supplemental appendix 3. Key characteristics of the interview participants are outlined in table 2.

**Table 2:** Key characteristics of the 124 interview participants

Characteristic		Diversity achieved
Doctor interviewees (n=92)	Medical career stages	11 Foundation training, 11 locally employed, three clinical fellows, six core training, 19 specialty training, seven specialty and specialist, 35 consultants
	Medical specialties	Anaesthetics, clinical genetics, emergency medicine, general medicine, genitourinary medicine, geriatric medicine, haematology, intensive care, neurology, obstetrics & gynaecology, occupational medicine, oncology, ophthalmology, paediatrics, pathology, psychiatry, radiology, rehabilitation medicine, renal medicine, respiratory medicine and surgery
Other staff interviewees		Educators, senior leaders, managers, human resource and occupational health specialists, chaplains, psychologists, Freedom to Speak Up Guardians, Guardians of Safe Working, well-being and equality, diversity and inclusion leads
Age range		23–68 years (mean=41 years)
Gender		71% female29% male
Ethnicity		73% White British and White other19% Asian8% Black, mixed race or other

### Four key findings, based on the realist analysis

There were four key findings, linked to four areas of programme theory, underpinned by 21 CMOcs (table 3). The four key findings from the programme theory were: (1) solutions needed to align with problems, to support doctor well-being and avoid harm; (2) doctors needed to be involved in creating solutions to their well-being problems; (3) doctors often did not know what support was available to help them with well-being problems and, even when doctors did know what well-being support was available, (4) there were physical and psychological barriers to accessing well-being support. Examples from the data were mapped to key findings (table 4).

**Table 3:** CMOcs with supporting evidence derived from the realist interviews with eight trusts

Number	CMOc	Supporting evidence (exemplar quote(s) in italics)
1	When doctors’ essential needs are not met at work (C), they may feel that their needs are not prioritised or cared about by the hospital trust (M) worsening their well-being (O).	“*You have to get the basics right first before you start your well-being because otherwise you undermine the well-being. [But] they're invisible to anyone in any sort of management position because people assume the basics are covered. Having paper in the printer, or paper that is accessible out of hours when we do lots of our printing, having hand towels to dry your hands, having somewhere to store your lunch, somewhere to change if you cycle to work, somewhere you can sit and have a coffee with a colleague [or] a room you can meet your trainees in if there’s a problem*”. Participant 707.D.
2	When doctors’ essential needs are not met at work (C), or the working environment hinders their work (C), additional stresses may be experienced (M), making it harder for doctors to care for their patients (O), leading to reduced job satisfaction (O) and worsening their well-being (O).	*“Our doctors’ office on the ward at present is a little cupboard. It has no windows, it has two computers, so you literally sit like next to each other, like, shoulder to shoulder […] we don’t have a workspace that’s conducive to good work. [And] that feels really frustrating, because it feels like that should just be a given. Like if you need to pick up the phone, you’re not [thinking] ‘Which phone can I use?’. Given so much of our job is being on the computer or being on the phone, it feels like there should be plenty of resources available for you to be able to do that. [It] feels completely broken [and] actually trying to support doctors with the job they have to do would go such a long way, way more than any support [and] resources for well-being”.* Participant 211.D.
3	When doctors are offered well-being solutions that are misaligned with the causes of problems (C), they perceive these offerings as tokenistic and superficial (M), causing them to disengage with well-being initiatives (O).	“*I feel it’s quite half-hearted. They're going, ‘Oh, look, there’s these things you can do. Aren't we nice?’. But they're not addressing the real issues of [the] workforce crisis, the hours [and] pressures being unsustainable, the rota gaps, all of those things that actually cause us strain and stress. And the attitudes of certain doctors who are bullies, it’s just done as a ‘Oh look, we've gone cold water swimming’*”. Participant 804.D.*“I don’t think petting a dog, or a donkey, or a parrot, is going to help with my problems, and I think to try and suggest that it does is embarrassing. I don’t want to go to a cake sale, I don’t want to have my feet massaged, I just want somebody to make my day-to-day job as efficient and easy as it can be. So, fix the IT, that would help with my well-being”*. Participant 314.D.
4	When doctors are offered misaligned solutions (C), they feel they are not being listened to or understood by leaders (M), resulting in feeling unvalued, frustrated and dissatisfied (O) and/or worsened well-being (O).	“*So often they've got their rather expensive but useless idea. So, I think every department will send a box, the little goodie bag for like well-being. Basically what was in the goodie bag was a teabag and a biscuit and some card or whatever. [It’s] really insulting [and] if they did that for every single person in the hospital, that must have cost quite [a lot of] money and just… annoyed people. You know, honestly, they got it all wrong, you don't need that. You're not preschool children. The other thing was that someone actually thought it was a good idea*”. Participant 214.D.
5	Doctors’ well-being is improved (O) when the work environment supports them (C) and allows them to carry out their role effectively (C) because their psychological needs for autonomy, relatedness and competence are satisfied (M).	*“In the department I'm in, we have enough juniors that you don't feel like your entire job is service. You get opportunities to learn. And because of that [it means] that one day where you're not going to be in, it’s not going to cause a department to collapse”.* Participant 413.D.*“For junior well-being, the consultant team well-being is really important, I think, because you learn from them, you take your cues from them, and they set up this culture”*. Participant 803.D.
6	When the working patterns and environment enable doctors to rest, eat and drink, and sleep appropriately (C), their physiological needs are met (M) and their well-being is improved (O).	*“There is an appropriate staff room [which] gives a place for you to relax, with mugs, with hot water supply, with tea supply. There are vending machines so you can get food if you need a snack. There’s a bed, with an actual mattress and easy access to sheets that you are allowed to sleep in, and there’s a computer, easily accessible. Which just means that I can have efficient, high output work without having to spend half an hour looking for a computer to log into to start working on”.* Participant 509.D.
7	When well-being solutions are linked to and tackle identified workforce issues (C) they are more likely to align with problems (O), have a preventative/proactive focus (O) and be considered favourably by doctors (O), because they are more likely to focus on addressing the underlying causes that impact on well-being (M).	*“[Having] the ability to talk through what you need and making reasonable adjustments makes an enormous difference to people and can potentially stop them from becoming totally overwhelmed. And that’s really powerful in terms of […] feeling connected to the people you work with and feeling held by your organisation. And then that kind of buy in and loyalty helps with staff retention and, you know, all those other challenges as an organisation”.* Participant 203.S.
8	When those responsible for doctors’ well-being improvement engage with doctors to understand the issues that they experience (C) and are supported by senior leadership to find solutions to well-being problems (C), they are able to make meaningful changes based on doctor input (M), so solutions are better aligned to the problems (O).	*“We went through a bit of a phase of slightly gimmicky well-being interventions which thankfully seem to have disappeared now. […] We’ve introduced some new systems. We’ve introduced some new people that have significantly reduced the amount of work that the junior doctors have to do, so they’re no longer being overwhelmed. And we’ve taken away the bleep [because] they were being pulled in 101 different directions. We’ve got someone who triages the bleeps and then just gives them the jobs [which has] had a huge impact and a lot of that has come from the junior doctors. They told us what the problem was and we’ve got a brilliant chief registrar who is leading that piece of work and is continuing to make improvements to the functioning of the hospital out of hours”.* Participant 204.L.
9	When senior doctors are supported to identify and make local changes to improve well-being (C) and have sufficient resources and authority to make changes (C), they may perceive that they have greater agency to make change (M) and develop local solutions that improve well-being (O) and create a positive local culture (O).	*“[At] the beginning of Covid, [we] arranged for a psychologist who does some [deanery] work so people could self-refer through to them because our [staff support line] wasn't very well set up. And at that stage, doctors were really hesitant to use the [staff support line]. And then [we did] a lot of promotion around the [staff support line], and we have noticed the numbers of senior doctors referring themselves has increased”.* Participant 202.D.S.
10	When doctors are not enabled to make local changes (C), they can feel frustrated and demoralised (M), worsening their well-being (O).	*“I think over the years, when you come up with ideas, if they get shot down, you reach a point where you think ‘It’s not worth it, can't be bothered. It’s just a waste of breath’. So eventually you just think ‘Well, what’s the point?’ [and] you think ‘Well, I'll put my energy and focus into other things where, actually, I know I'm going to get good, positive results’”.* Participant 414.D.*“I think it’s very control and command and hierarchical, and it doesn't give people to space to work… independently, and be creative, because of this multilayered thing you've got to go through to get things signed off and… yeah, it’s frustrating, I find it frustrating”.* Participant 703.S.
11	Solutions are likely to be better aligned to the problems that influence well-being (O) when those initiating changes/interventions have time allocated to engage with feedback and design interventions (C) because they are able to think through the problem and identify appropriate solutions (M).	*“You know, we hear pockets of team activities, that somebody has an interest so they take the lead, certainly in areas such as critical care, ED, where you’ll get an individual who has a passion, so they try and do that team level support. [But] from what I’m hearing, everybody is just too busy just trying to survive their day, and that ability, to do some blue sky thinking, or to take time out to put some mechanisms in place, I don’t believe there’s much opportunity to do that”.* Participant 711.S.
12	When people do not have sufficient time allocated to engage with and address feedback (C), then they are less able to think through the design interventions and potential implications (M), so solutions are more likely to have unintended consequences (O).	*“We spend two min talking about the problem, then jump to a solution, and then work out how we're going to implement the solution. And what I think increasingly I'm finding is that if you can fit in there [and as a group, say] ‘Let’s stop thinking of solutions, let’s really define the problem’ and again, that’s something for leadership support, then you're much more likely to find the right solution”*. Participant 202.D.S.
13	When there are clear and confidential processes for raising and resolving concerns and problems (C), doctors feel heard and valued (M), supporting their well-being (O) and/or positive perceptions of the organisation (O).	*“I mean, our department has [these] support forums that will happen every now and then, and we can anonymously raise our concerns and frequently we have [a] workplace championship meeting, so we can discuss […] any concerns. Fortunately, I never had a very bad encounter, [but] I know that when something is being raised, that is being addressed. I know a few occasions where that has been addressed and it has been resolved”*. Participant 516.D.
14	When processes for raising issues have repercussions for doctors (C), they feel frustrated, unsafe and/or victimised and also not listened to (M), so well-being is worsened (O) and/or doctors disengage from processes for raising issues (O).	*“Before starting, I heard from people ‘Don’t exception report because then they kind of you know, they just write a response basically saying that you’re incompetent, and it’s your own fault that you’re having to stay overtime’. [And] my supervisor gets a report when I exception report as well [so] I’m not getting anything out of doing this, you know, it’s just that I’m… painting a target on my back [as] either troublemaker or lazy doctor who can’t, you know, do [their] job properly”.* Participant 610.D.
15	When there is not clear communication about the support available to doctors who experience poor mental health or well-being (C), doctors feel unclear about where to go/how they might be supported (M), so are less able and willing to seek support when needed (O).	*“And then there is kind of like a [directory] of available things. But I find it quite difficult to navigate in terms of it’s like all resources for all staff within [the trust and] that doesn’t just cover [my area], and it doesn’t just cover doctors, and it can be hard sometimes to figure out is that [a resource] that’s actually useful to me. And then if it is does it work in times that I’m not [working and] how do I access it? Do I have to make a phone call and it’s going to be a half an hour hold time or, you know, things like that. It feels like there are a lot of steps to actually finding the support you need”.* Participant 211.D.
16	When doctors have a clear sense of who or where to go to for support (C), they feel secure knowing support is available if needed (M), and are more likely to seek support if needed (O).	*“When we have our meetings with our educational supervisors, or clinical supervisors, there’s a checkbox they have on our form which asks ‘Are you aware of the well-being services?’ or ‘Who you should speak to if there are any issues?’ So I think there’s a well-being service within the hospital, and if there were any issues I could contact my educational supervisor, and he’s also said that to me before as well, so yeah”.* Participant 513.D.
17	When doctors have supportive relationships with their colleagues (C), others can recognise when the doctor is not themselves (M) and offer support to them (O).	“*I've found my experience with my educational supervisor has been fantastic. He’s very attentive, and he actually realised I was struggling before I even realised I was struggling, because he’s got that experience of working with new doctors. I don't know how that goes as you work your way up the ranks but I think at the beginning they want you to adopt a good work-life balance and good well-being from the start”.* Participant 811.D.
18	When solutions are not designed with doctors’ working patterns and environments in mind (C), doctors are less likely to engage with offers (O), because they perceive these to be inconvenient and inaccessible (M).	*“[The well-being weeks run by the well-being team are] probably not positive for doctors and nurses, and that’s because a lot of the stuff […] would be on at 10 o’clock in the morning. There’s an opportunity to do yoga [but] the nurses absolutely can’t leave the ward and the doctors are all stuck on a ward round, so that’s “fab” [said ironically]*. *So it actually felt like there was this offer […] that we then couldn’t go to, which almost felt like ‘You’re not important. What we actually care about is the people in offices who are able to do that’”.* Participant 302.D.S.
19	Doctors feel safe to engage with support solutions (O), when there are no perceived negative repercussions from accessing them (C), because this minimises the potential risk of accessing support (M).	*“[The well-being] support thing’s really good, and it’s quite nice that it’s not directly linked with the hospital as well, because… there’s still a stigma around it, so you don't want to be like, you know, walking around and seeing someone that has helped you around the hospital, because it probably feels like they're coming into your space”.* Participant 811.D.
20	When support offerings are designed to protect confidentiality (C), doctors are more willing to access them (O) because they feel safe to do so (M).	*“I have in the past accessed PHP [an external NHS Practitioner Health Programme], and a mindfulness programme. All of which I thought were very useful, and which, as far as I'm aware, remained confidential, and didn't get discussed with anybody in my department, or training leads or anything like that”.* Participant 110.D.
21	When doctors receive incomplete and/or insufficient well-being support (C), they may feel frustrated with the service provided (M) and/or regret engaging with well-being services/solutions (O) and/or may experience worsened well-being (O).	*“He was like 'We can only offer four sessions' [of psychological therapy] because they have a waiting list. And I thought ‘Gosh, like, just as I'm getting to the peak of what I'm, you know, feeling, and it just ends’. He himself said ‘A lot of people find it more beneficial when they have a few months of this'. And so four sessions just wasn't really going to cut it, so I kind of just left it, really. I don't know who thought that was a good idea, to be honest”.* Participant 415.D.

Where a single quote can cover the complete CMOc well, only one quote is provided. After the quote the participant identifier is given, which comprises a unique number plus a suffix.

CMOcs, context-mechanism-outcome configurations; D, doctor; DS, joint doctor/support role; L, leadership role; S, support role.

**Table 4:** Key findings mapped to CMOcs and examples of positive and negative support interventions sourced from data collected within the eight trusts

Finding	Examples from the data
**Solutions needed to align with problems, to support doctor well-being and avoid harm**	
Misalignment between doctors’ essential needs at work (physiological, physical environment, social/belonging, safety) and the work environment.This caused harm through not feeling cared about, as well as the physical impact of not being able to function well.*CMOcs 1, 2*	Encouragement from leaders to eat well was felt to be misaligned with the canteen food available. Being asked to cover shifts when scheduled to take a day off did not meet the doctors’ needs for rest and recuperation, causing harm by making them feel uncared for. Poor office equipment and a door that did not lock made work difficult and detracted from a sense of belonging and being valued. Pressure to remove a coffee break after ward round (since this was deemed ‘not work’) made doctors feel undervalued.
Misalignment was frequent between the causes of well-being problems, as perceived by doctors, and the solutions offered by trusts.Misaligned solutions often did not address well-being problems and were viewed as tokenistic or misguided. This caused harm through feelings of frustration, dissatisfaction and being undervalued.*CMOcs 3, 4*	Problems of overwork and poor IT systems were not addressed by ‘solutions’ such as gifts of tea bags, yoga, animal visits and mindfulness. Doctors felt unheard, misunderstood and insulted by the offer of resilience training. Doctors felt irritated by ‘well-being emails’, which sometimes felt generic and unhelpful, while adding to already high workloads.
Doctor well-being was improved when there was better alignment between their working environment and their essential and psychological needs.*CMOcs 5, 6*	A ‘midnight huddle’ with hot soup provided sustenance, social support and made doctors feel cared about. A designated ‘quiet’ chair with headphones in the rest space for a doctor to be able to show they did not want to be disturbed. Provision of beds for on-call staff.
Well-being solutions were more likely to align with problems when leaders linked well-being with patient safety and workforce issues.*CMOc 7*	Workforce issues (eg, recruitment, retention) were used to make business cases for well-being initiatives. Staff well-being was a default agenda item (alongside other workforce issues) at senior management meetings—and other meetings at multiple levels/within different directorates. The well-being team created an explicit strategy to generate ‘buy in’ from senior leadership on well-being issues.
**Doctors needed to be involved in creating solutions to their well-being problems**	
Initiatives that were doctor-led or doctor-informed were more likely to be effective.Understanding the problem and potential impact of solutions was needed, through engagement with those directly affected.Senior doctors often had a significant influence on the working environment and culture at a local level.*CMOcs 8, 9, 10*	A bleep coordinator for night shifts was introduced as a result of junior doctor/Guardians of Safe Working feedback. Relaxer chairs for junior doctors were bought using funding from senior doctor lecturing. Senior doctors actively promoted a supportive/positive well-being culture, for example, introducing a coffee break after the ward round for doctors and medical students. Doctors were encouraged to use exception reporting to identify concerns (eg, not getting breaks), which were followed up supportively by senior staff. Meetings were introduced where doctors were encouraged to discuss concerns and how these could be resolved.
Staff needed to feel able to make meaningful change that addresses problems identified. They also needed time to think through solutions and potential impacts, to avoid harm.Excessive bureaucracy could block efforts to make change.Without supportive conditions, implementing change required a champion, who may face resistance.*CMOcs 11, 12*	Doctors reported that they were allowed to attend a well-being activity but not given the autonomy to choose their own well-being solutions. An extra day off for staff was given, but doctors were unable to take it due to understaffing and limited involvement of rota coordinators. Funds were allocated for cocoon-style chairs to support on-call doctors to sleep but by the time the purchase was agreed, the funding had been spent elsewhere.
Clear processes were needed for doctors in different roles to safely raise/identify issues and for this feedback to be considered and acted on by leaders.Harm could occur if feedback was not dealt with appropriately or if doctors experienced negative repercussions from raising issues.*CMOcs 13, 14*	Multiple feedback mechanisms provided, for example, exception reporting, meetings with junior doctors, junior doctor forum, department meetings, surveys. Exception reporting was sometimes interpreted negatively, for example, to suggest doctors were being slow or not prioritising effectively. This caused harm and discouraged doctors from raising subsequent issues. Similarly, reports to Guardians of Safe Working could make doctors feel like a troublemaker or lazy. Yet in another trust, such reports led to additional medical staffing. In one trust, the Freedom to Speak Up Guardians reported that felt bullying and harassment was not their business and, in another, they did not respond to emails from doctors.
**Doctors often did not know what support was available to help them with well-being problems**	
Doctors were often unaware of the support services available that could help with specific problems. Few (if any) people had a comprehensive overview of all support available.This could be exacerbated for those outside of formal training posts.Sometimes the amount of information could feel overwhelming.*CMOc 15*	Online information was often disorganised and scattered over many platforms. Sometimes, even when doctors used phrases such as “I am feeling burnt out” or “I am really struggling”, their supervisors did not signpost/refer to support services. Occupational health roles seemed to vary by trust, with some more involved in staff well-being than others.
Doctors did not need to know about all support available but did need to know where to go when support is needed (and feel safe to do so, see also CMOcs 19 and 20).This required those in supervisory/line management/leadership roles to be able to signpost to support.*CMOc 16*	Some well-being intranet pages and staff provided a clear and direct line to support options. Toilet door posters seemed effective for communicating the options available. A checklist for education supervisor meetings was introduced, including the item ‘knows how to access well-being support’. Supervisor training was provided for how to signpost to support.
Doctors did not always recognise when they needed extra help or support.Colleagues needed to be attuned to the well-being needs of others. This was more challenging for doctors rotating frequently or working transiently.*CMOc 17*	Educational supervisor meetings were scheduled with a broader ‘check in’ focus, not just a training focus. A clinical psychologist was embedded within the clinical team. Daily team check-ins, led by the registrar, to ensure that everyone is okay and managing their workload for the day. Consultant WhatsApp groups. When doctors worked across multiple sites while on-call, there was limited opportunity to get to know people and access peer support or intervention.
**There were physical and psychological barriers to accessing well-being support**	
Doctors reported that problem-focused solutions, for example, counselling, were often not accessible or timely, resulting in delays to accessing services and increased risk of worsening well-being/ill-health.Support needed to be physically accessible to doctors (but those designing may not know the constraints for doctors).*CMOc 18*	Doctors were unable to access a mental health triage phone call because the only available times were when they were on the ward. Doctors were not always able to self-refer, for example, to occupational health, plus there were often very long waiting times. Interventions designed to be preventative, such as yoga and well-being walks, were often unavailable to doctors due to their working patterns.
Support options needed to feel ‘safe’ so that doctors felt they could access them.Improved psychological safety allowed people to raise issues earlier, so they could get resolved before becoming more serious.Stigma remained around mental health among doctors, which could be influenced by cultural expectations, for example, for International Medical Graduates.*CMOcs 19, 20*	Confidentiality of accessing support was compromised if doctors had to explain why they were leaving the ward. Doctors felt that seeking support could impact their progression, especially if the clinical supervisor was both the gatekeeper to support and to career advancement. External support services were seen as ‘safer’ by some doctors, for example, Practitioner Health Programme. Modelling by senior staff was felt to impact on the acceptability of seeking support.
When support services for mental ill-health were poorly designed/implemented, this could create further problems and act as a barrier to accessing further help.*CMOc 21*	One doctor was offered limited sessions of psychological therapy, such that the issues identified were not resolved within the timeframe. Delays in referral processes and/or service delivery were frequently experienced, for example, occupational health. When nothing changed after signalling for support (eg, saying I am feeling burned out), doctors felt it was pointless and a waste of time. Occupational health support was sometimes perceived as more focused on steps back to work/patient safety, rather than understanding the causes of workforce ill-health.

CMOcs, context-mechanism-outcome configurations.

#### Solutions needed to align with problems, to support doctor well-being and avoid harm (CMOcs 1–7)

Participants often reported misalignment between doctors’ essential needs at work and what the work environment provided, which impacted negatively on well-being (see table 3, CMOc 1 and 2 quotes). The resulting harm occurred both through the sense of not feeling cared about, as well as the physical impact of not being able to function well and/or having to deal with additional problems that create unnecessary stress. These essential needs included physiological needs (eg, rest/breaks, food, hydration, toilet breaks, sleep); physical environment needs (eg, space to work, functioning IT systems, space to put belongings, rest/break spaces), social/belonging needs (eg, team connection and belonging, feeling recognised and valued) and safety needs (eg, physically and psychologically safe work environments, staffing levels). For example, many doctors reported being unable to access hot food at night or a quiet space to work or rest during shifts. Sometimes, staff shortages meant they had to work extended hours without a break.

There was also misalignment reported between the solutions offered by trusts and the problems doctors perceived as affecting well-being (see table 3, CMOc 3 and 4 quotes). This meant that well-being was not improved by these offerings and was often viewed as tokenistic, leading to reduced faith in trusts and harm through feelings of frustration and not being valued. This dissatisfaction could worsen well-being. For example, if well-being ‘problems’ were seen to arise from overwork and an inability to deliver optimum patient care due to time pressures and poor equipment, then ‘solutions’ such as gifts of tea bags, yoga and animal visits were not seen as helpful. These initiatives neither solved nor addressed underlying problems, and doctors reported feeling that their trusts did not listen to, understand and/or care about the workplace challenges they faced or the negative impacts on patient care. The following quote illustrates this well (participant identifiers are explained in table 3):

I don’t think petting a dog, or a donkey, or a parrot, is going to help with my problems, and I think to try and suggest that it does is embarrassing. I don’t want to go to a cake sale, I don’t want to have my feet massaged, I just want somebody to make my day-to-day job as efficient and easy as it can be. So, fix the IT, that would help with my well-being*.* Participant 314.D.

Participants explained that well-being solutions were more likely to align with problems, have a preventative/proactive focus and be considered favourably by doctors when trusts (eg, senior leaders) linked well-being to high-level priorities such as patient safety and workforce issues (eg, staffing numbers, recruitment and retention metrics, sickness absence) (see table 3, CMOc 7 quotes).

One example of a positive intervention, relevant to this finding, was a ‘midnight huddle’, whereby the problem of many junior doctors feeling isolated and unsupported in their work at night was addressed by an opportunity for support, social connection and sustenance (eg, food) (see table 4, CMOc 5 and 6 examples).

#### Doctors needed to be involved in creating solutions to their well-being problems (CMOcs 8–14)

Initiatives that were either doctor-led, or closely linked to doctor feedback, were perceived as more likely to be effective in improving doctor well-being (see table 3, CMOc 10 and 13 quotes). To create effective solutions, there needed to be sufficient understanding of both the problem and the potential impact of solutions, through meaningful and regular engagement with those directly affected. However, this did not always occur, as this quote illustrates:

I think over the years, when you come up with ideas, if they get shot down, you reach a point where you think ‘It’s not worth it, can’t be bothered. It’s just a waste of breath’. Participant 414.D.

Senior doctors were felt to have a significant influence over local cultures and well-being (both positively and negatively), if they felt able to identify and make local changes that supported the medical workforce in their team/department/specialty. For example, one senior doctor introduced a post ward round coffee, with the intention of helping more junior colleagues to feel a greater sense of belonging.

Well-being leaders and doctors reported needing the ability to make meaningful changes that addressed the problems they had identified. However, this depended on having sufficient time to think through solutions and their potential impacts, without which solutions could miss the mark and cause more harm (see table 3, CMOc 11 quote). It also depended on there not being excessive bureaucracy to block change efforts. Without supportive conditions, implementing change required a champion to push it through, but this person often faced resistance, and in unsupportive contexts the resulting change risked being dropped, for example, if the champion took on other roles/responsibilities or left.

There was a perceived need for clear processes through which doctors in different roles could safely raise/identify issues without fearing or experiencing negative repercussions, and for this feedback to be considered and acted on by those responsible for well-being. Harm could result if feedback was not dealt with appropriately or if doctors alone were expected to solve their own problems. If doctors raised issues and these were ignored, they could feel demoralised and disengaged. Similarly, if doctors experienced blame or other negative repercussions from raising issues (eg, exception reporting, which is a mechanism through which doctors in training can report extra hours worked), well-being could be worsened (see table 3, CMOc 14 quote). For example, the Freedom to Speak Up Guardians (a role introduced as an additional route for healthcare workers to raise concerns) at one trust said that bullying and harassment was not their business (because they focused on ‘patient’ issues only, failing to acknowledge a connection between the two), and at another they often did not respond to doctors’ emails.

An example of a positive intervention identified, relevant to this finding, was the introduction of a ‘bleep coordinator’ for night shifts in response to feedback from junior doctors and the trust’s Guardian for Safe Working (see table 4, CMOc 8, 9 and 10 examples). A bleep (or pager) is still often used in hospitals, especially if wifi connection is patchy, as a means to contact a doctor on duty to request their assistance. On night shifts, where doctors may cover multiple wards, individual doctors may receive a high volume of bleeps at the same time. Thus, having a bleep coordinator to monitor the bleeps centrally and allocate/coordinate/prioritise the work was a welcome intervention.

#### Doctors often did not know what support was available to help them with well-being problems (CMOcs 15–17)

Most doctors were not aware of which support services were available within trusts to help them when experiencing difficulties (see table 3, CMOc 15 and 16 quotes). Few people (if any) had a comprehensive overview of all the support available. A clear communication strategy about well-being support was often lacking, leading to an overwhelming amount of information that made it hard to identify appropriate support when needed. This seemed to be exacerbated for those outside of supervisory roles or formal training posts (eg, specialty and specialist doctors, locally employed doctors).

I find it quite difficult to navigate… it doesn’t just cover doctors, and it can be hard sometimes to figure out is that [a resource] that’s actually useful to me. Participant 211.D.

Participants felt doctors did not need to know about all support available, but they needed to have a clear sense of who/where to go when support was needed (and feel safe to do so). This required supervisors/line managers/leaders roles to be able to signpost others, but many doctors reported that supervisors were also unclear about support options.

Participants noted that doctors did not always recognise when they needed extra help or support, in which case others (peers, seniors) needed to be attuned to the well-being needs of colleagues. This was more challenging for doctors rotating frequently or working more transiently, such as locum staff.

An example of a positive intervention identified, relevant to this finding, was posters on the backs of toilet doors that clearly showed which options were available depending on the severity and type of problem (see table 4, CMOc 16 examples). The posters were visually clear and simple, making them memorable.

#### There were physical and psychological barriers to accessing well-being support (CMOcs 18–21)

Even when a support option had been identified and made available, participants reported physical barriers to accessing support (see table 3, CMOc 18 quote). In many cases, the support offered was not accessible during normal working patterns, either because doctors were unable to leave the ward, or due to the lack of a confidential space to take telephone calls. This problem could be linked to the fact that those designing interventions were often not doctors or were distant from the working conditions of frontline doctors.

I’ve done a triage appointment before like in a corridor at work, and talking about how my work causes me anxiety… I’ve done it, actually, I sat on a toilet. I did… did my mental health triage appointment, sat on a toilet on a ward, because the appointments were Monday to Friday 9 to 5. Participant 418.D.

In addition to physical barriers, doctors reported psychological barriers to accessing support (see table 3, CMOc 19 quote). This included concerns about how this might be perceived, especially by senior colleagues. This seemed to be linked to the local workplace culture and professional culture of medicine, in which there is still stigma around mental health. It could also be affected by doctors’ backgrounds. For example, one participant who was an International Medical Graduate doctor did not realise that well-being support is offered at work in the UK, as this may be less typical internationally.

When support services for doctors’ mental ill-health were poorly designed or implemented, this appeared to create additional problems. One example was when a doctor was only able to access a very limited number of sessions of psychological therapy, such that they started to open up about an issue but did not have sufficient supported time to resolve it.

Examples of positive interventions identified, relevant to this finding, were positive modelling by senior staff, which changed perceptions about the acceptability of help-seeking; and signposting to support outside of the organisation, which was seen as psychologically ‘safer’ (see table 4, CMOc 19 and 20 examples).

**Figure 1:**
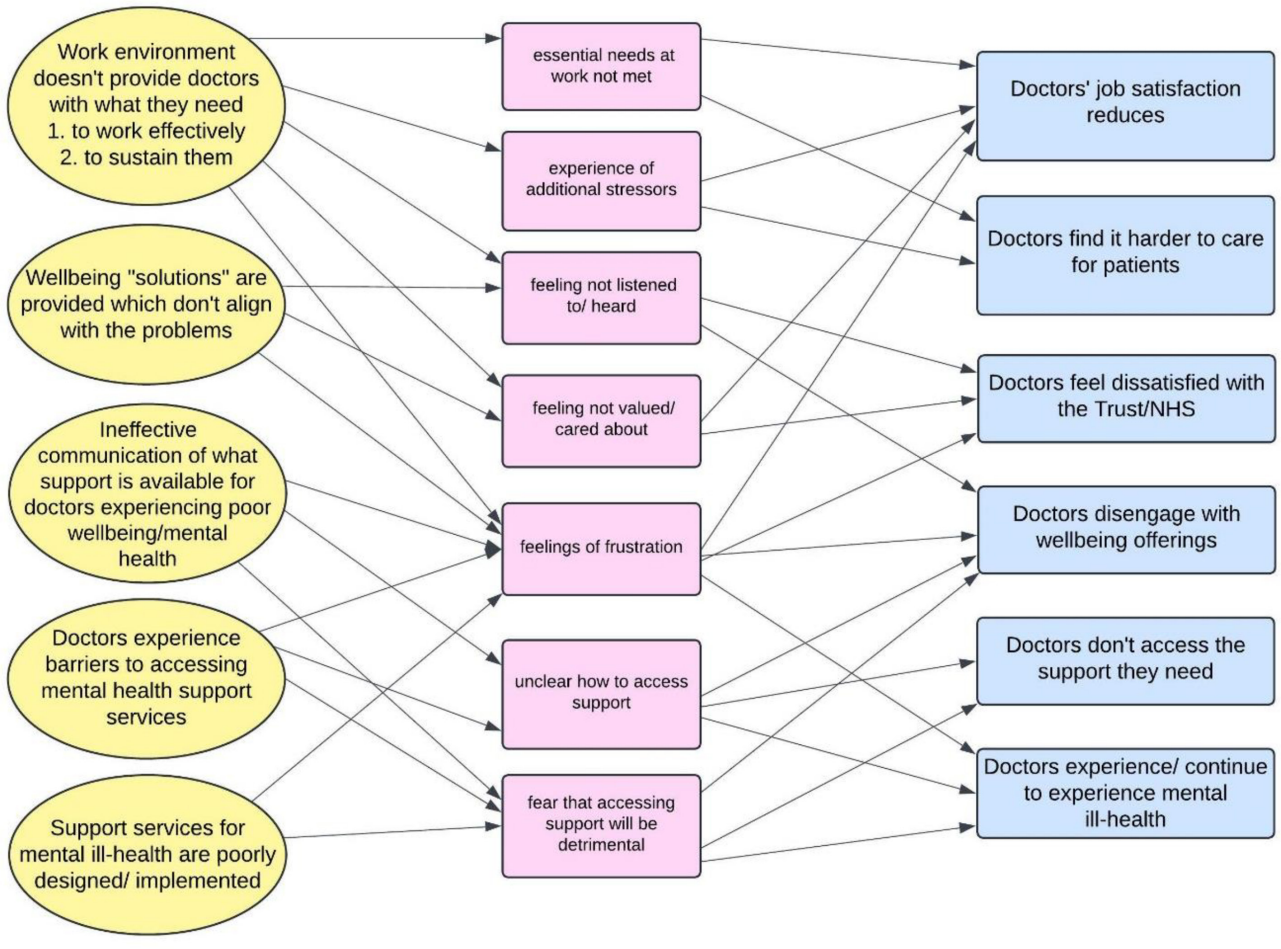
Graphical representation of final programme theory, focussing on the negative aspects of outcomes since these were more prevalent in the data. NHS, National Health Service.

The final programme theory, reflecting all four findings, is represented graphically in figure 1.

## Discussion

The aim of this research was to work with, and learn from, eight diverse hospital settings to understand how to optimise strategies to improve doctors’ well-being and reduce their negative impacts on the workforce and patient care. The key findings were that well-being solutions needed to align with problems, that doctors needed to be involved in creating solutions, that doctors often did not know what support was available and that doctors experienced physical and psychological barriers to accessing support. Important contexts such as work environments not meeting doctor needs, lack of alignment of problem and solution, poor communication of support options, barriers to accessing support and poorly designed/implemented support interventions drove mechanisms such as not feeling heard or valued, feeling frustrated, feeling confusion about how to access support and feeling fearful of the consequences of help seeking, which led to outcomes such as reduced job satisfaction, finding it harder to care for patients, feeling dissatisfied with employers, disengaging from well-being offerings and continuing to experience well-being problems. Doctors are mandated to ‘first, do no harm’ to their patients, and in this paper, we argue the same consideration should be extended to doctors. This study’s findings, and the positive and negative examples from the data, will enable health services to avoid further harm.

Our research extends knowledge by offering an explanatory theory of whether and how existing interventions are working. It builds on our realist synthesis that reviewed interventions to tackle doctors’ and medical students’ mental ill-health and its impacts on the clinical workforce and patient care.^24 25^ Our study corroborates those findings and adds important novel insights, for example, that interventions that are misaligned to the issues they are trying to address are not just ineffective but can be harmful—contributing to doctors’ feelings of physical and emotional isolation.

These findings contribute to a growing body of evidence that recognises that poor well-being of healthcare professionals is often the result of organisational problems (eg, high work demands, long working hours, low job control, top-down pressure, unrealistic expectations) rather than only individual ones.^724^,^26 30^ An important implication of this is that individual-level well-being interventions (such as resilience training) are likely to be ineffective, at least in isolation, and that organisational interventions are also needed to prevent and reduce poor well-being at work.^42^

Previously, Gordon *et al* used multiple multidimensional transitions theory to study how doctors’ transitions during COVID-19 impacted on social and cultural aspects of well-being.^33^ They found three factors contributing to well-being: being heard (eg, by colleagues asking how they are); feeling valued (eg, through provision of rest spaces) and being supported (eg, through regular briefing). Our realist evaluation highlights the nuanced ways in which participants associated a lack of being valued with their essential needs not being met. Interestingly, both studies highlight rest spaces as an important intervention, despite a recent scoping review highlighting a lack of research investigating their effectiveness.^43^

Our realist evaluation supports other research that concludes there is an urgent need to rebalance and refocus work efforts on multilevel systems approaches that take account of the often-conflicting interests between serving patients and protecting staff well-being.^26 44^ This will require broader definitions that include interventions that improve the work environment and systems. Individual-focused solutions are only effective within enabling environments.^45^

Workforce well-being has often been seen by organisational leaders as an optional extra, rather than critically interlinked with organisational performance. Our research adds to literature that positions staff well-being as critical for tackling workforce issues (eg, recruitment, retention), which, as reported by the Quadruple Aim of healthcare, is essential for achieving the other three aims: improving patient experience, enhancing population health and reducing costs.^2^ Participants reported that ineffective well-being interventions can be harmful, consistent with prior research.^22^ Our research also surfaces examples where small changes, if properly developed and implemented, create positive results.

### Strengths and limitations

We identified three key strengths. First, realist methodology was uniquely well placed to accommodate the complexity of topic and multiple settings. Second, the relatively large sample size and realist approach enabled us to make stronger claims about transferability of findings than we might otherwise have felt able to do. Although the data were collected in England, we anticipate that the causal explanations (in the form of CMOcs) may work similarly in other countries. For example, a US study of resilience interventions highlighted potential negative impacts.^22^ Previous work also concluded there are more similarities than differences in well-being experiences between healthcare professionals,^46^ so the key principles might be tailored for other groups within hospital settings. Third, the involvement of researchers and stakeholders with different disciplinary/professional backgrounds ensured that rich perspectives informed the research. As with all research, there were limitations as well. Although we sampled for diversity, the eight trusts were self-selecting since they agreed to be involved. Therefore, the findings may not represent all trusts in England. There are also limits to transferability. We anticipate that further research would be required to tailor the findings to primary care, mental health or social care settings. Finally, there were some important perspectives that we were unable to engage in the research, most notably those from finance teams, so this is a priority for future research.

### Implications for policy and practice

First, it is important for employers and policymakers to understand that, despite positive intentions, poorly designed well-being interventions can cause harm; in fact, many are doing so now. Therefore, new policies and interventions require careful planning and evaluation. Second, it is increasingly clear that, unless organisations prioritise workforce well-being, they will not achieve the positive workforce, finance and patient outcomes that they seek. Healthcare employers need to recognise doctor well-being as essential for high-quality health service delivery and prioritise it as an integral part of the trust’s work. While initiatives involving doctors are more likely to be effective, importantly, this does not mean that solving well-being issues at work should be solely doctors’ responsibility. Indeed, seeing it as such would miss opportunities to address the structural causes of the problems. A commitment to improvement requires a shared vision and breaking organisational silos, which will require critical allies.^47^

Third, our research identified many ineffective or harmful interventions that could be stopped, which would reduce costs, improve well-being and unlock other positive workforce and patient care outcomes.^2^ Similarly, many of the positive examples identified were inexpensive to deliver, especially in comparison with the huge cost of staff sickness and attrition. Finally, healthcare employers would be wise to invest time in proactively supporting doctors’ well-being (eg, improving the workplace, increasing personal resources), as well as resolving problems after they arise.^31^ Support options need to feel 'safe' and accessible, without negative repercussions. Improving psychological safety would allow issues to be raised and resolved earlier (ie, when problems are still minor).^12^

### Implications for future research

Realist approaches seem particularly well suited to this topic. Future research could test our programme theory in other countries or settings (eg, primary care, mental healthcare, social care). Specific implications for early career health professionals could be explored, to extend understanding of how workforce well-being interacts with education. Finally, health economic studies of well-being interventions (ideally at systems level) are urgently required, since small investments could have far-reaching positive impacts.

## Supplementary material

10.1136/bmjqs-2024-017698 online supplemental file 1

## Data Availability

No data are available.
