# Supplementary material for: Support for hospital doctors’ workplace well-being in England: the Care Under Pressure 3 realist evaluation
Source: BMJ Qual Saf. 2025 Apr 11;34(7):e017698. doi: 10.1136/bmjqs-2024-017698 (PMC12229071; doi:10.1136/bmjqs-2024-017698)
Supplement: online supplemental file 1 [file bmjqs-34-7-s001.pdf]

Appendix 1. Criteria used for selection of NHS trusts.

Domain	Data source	Particular focus
Quality of care	Care Quality Commission (CQC) inspection ratings	'Well-led' and 'Overall' ratings.
Staff satisfaction	NHS Staff Survey 2021	Q11a – 'My organisation takes positive action on health and wellbeing' (% agreeing)<br>Q22a – 'I often think about leaving this organisation' (% agreeing)
Supportive environment	General Medical Council (GMC) National Training Survey 2022	Overall satisfaction<br>Supportive environment<br>Workload.
Socio-economic status of population served	English Indices of Deprivation 2019 Research report	Deprivation ratings of the areas in which Trusts were located.
Other characteristics	Trust websites<br>Office for National Statistics	Size (number of beds/sites/staff)<br>Location e.g. rurality<br>Demographics of population served

Appendix 2: Topic guide

Introduction to the interview

Thank you for taking part in this interview. The purpose of this research is to evaluate existing approaches to supporting doctors' well-being at work within the trust. When we talk about 'supporting doctors' well-being', we are referring to a wide range of potential interventions, approaches or initiatives, including individual, group, team, organisational, and national changes, not just individual ones. For example, this might include policies; changes to working conditions, environments or practices; services for well-being; stress management training; counselling services etc. Similarly, when we say 'well-being' we recognise that this can refer to a wide range of experiences, from serious mental ill-health problems to positive work experiences.

We won't be asking specifically about your personal experiences relating to mental health, so please do not feel that you have to share these, but also please feel free to draw upon them if you want to. We'll be focusing on the trust's support for doctors, with the aim of understanding what is working well (or not) and why, including how this varies for different people or in different settings.

We will use our research findings to make practical and realist recommendations that will benefit doctors in the NHS. Ultimately we are trying to understand how to optimise the approaches that are already being offered by the trust (and where needed), suggest alternative options.

Once recording initiated

Opening	**1. Are you doing this interview during your working hours?**
	**2. Which hospital(s) do you work at within the trust?**
	**3. What interested you in taking part in the study?**
Perceptions of well-being	**4. What does "well-being" mean to you?**<br>a. For example, for some people it might mean finding meaning and satisfaction in their work, while for others it might relate more to reducing mental ill-health.<br>b. Why is doctors' well-being important?
	**5. What kinds of things influence doctors'/your well-being at work? Why?**
Organisational culture	**6. What's it like working here? Why?**
	**7. How much of a priority is doctor well-being within the trust?**<br>a. Could you give me an example to illustrate that?<br>b. Do you think other people in the trust would agree? Why/not?<br>c. How does your trust monitor if doctor wellbeing is improving/ declining?
Well-being strategy	**8. Who are the people responsible for well-being in your organisation?**<br>*For those with well-being roles:*

	a. How did you come to be the well-being lead/take on this well-being role?<br>b. What interests you about well-being and why?<br>c. What is included within your well-being role?<br>d. What support and resources (including budget, what committees they sit on, senior buy-in and back up) do you receive for your role?<br>e. Please tell me about any protected time you receive for the role.<br>*For those not in well-being roles:*<br>f. How do those in well-being roles find out about what you need?<br>g. How could they find out about your needs?<br>h. How do you get to find out about what changes they make?
	Filter question to see if applicable: ***What knowledge do you have of the trust's strategy to supporting doctors' well-being at work?***<br>**9. How would you describe the trust's approach to supporting doctors' well-being at work?**<br>a. Can you give any examples?<br>b. For example, it might include policies; changes to working conditions/ environments/ practices; services for well-being; stress management training; counselling services etc.<br>c. Do you know how any of these interventions are funded?<br>d. To what extent were staff involved in designing or deciding the approach?<br>e. Please give me an example of informal things happening in the workplace to support well-being.
Well-being interventions	**10. What kinds of things have worked well to promote better experiences at work for doctors?**<br>a. Can you give any examples?<br>b. What it is about it that helps doctors' well-being?<br>c. Which doctors might benefit from this do you think?<br>d. Can you tell me about any combinations of interventions that (might) work well together in supporting doctors' well-being?
	**11. What would happen here if a doctor was experiencing difficulties with their mental health?**<br>a. Can you give any examples?<br>b. What would happen if a doctor was having difficulties with their physical health?<br>c. If a doctor was experiencing problems in their personal life, would there be any kind of help for that?

	d. What approaches have worked well to help doctors who are struggling with their mental health/well-being?
	**12. Please tell me about any approaches that you don't think were helpful in supporting well-being?**<br>a. Can you give any examples?<br>b. What was it about it that wasn't helpful?
	**13. Please tell me about anywhere else that doctors might go to get support outside of the trust?**
Closing	**14. If you had a magic wand, what would you do to ensure workplace well-being? Why?**<br>a. What barriers are there to making the changes that you have suggested? [e.g. financial or time costs]
	**15. Is there anything else you'd like to add?**

Appendix 3: Key characteristics of the eight trusts

Trust	Region	Rural-city	Number of staff	Deprivation
1	South	City	>20,000	High
2	South	Rural	5-10,000	Low to moderate
3	South	Rural	5-10,000	Mixed
4	North	City	5-10,000	Very high
5	South	Rural	< 5,000	Mixed
6	Central	City	10-20,000	High
7	North	City	10-20,000	Very high
8	South	Rural	5-10,000	Moderate to high
